# A Community-Based Investigation of Stigma Toward Individuals Receiving Methadone Maintenance Treatment in China: A Randomized Case Vignette Study

**DOI:** 10.3389/fpsyt.2020.601266

**Published:** 2020-11-25

**Authors:** Qijian Deng, Maorong Hu, Fang Yu, Qiaosheng Liu, Wei Hao, Qiuxia Wu, Tao Luo

**Affiliations:** ^1^Department of Psychiatry, Second Xiangya Hospital, Central South University, Changsha, China; ^2^Department of Psychiatry, The First Affiliated Hospital of Nanchang University, Nanchang, China; ^3^Department of Psychology, Jiangxi Mental Hospital, Nanchang, China; ^4^Department of Social Medicine and Health Management, Xiangya School of Public Health, Central South University, Changsha, China

**Keywords:** stigma, methadone maintenance treatment, MMT, China, public attitudes

## Abstract

**Background:** Even though methadone maintenance treatment (MMT) is effective and widely recommended, it is widely misunderstood and stigmatized. This study aimed to explore people's attitudes and beliefs toward MMT, and stigmatization of MMT patients in China.

**Methods:** This randomized, vignette-based study enrolled 1,066 household respondents (552 males and 514 females, response rate is 88.83%, 86.00% in vignette 1 and 91.66% in vignette 2) from two communities in China. Respondents initially completed questionnaires on sociodemographic characteristics and their knowledge about methadone and MMT. They were then randomly assigned to vignette 1 (described a patient receiving MMT) or vignette 2 (described a patient receiving amlodipine treatment). The labeling, stereotyping, and social distance toward the individual described in the vignettes were evaluated.

**Results:** In these two vignettes, respondents showed a significantly higher level of stigma and discrimination toward the patient receiving MMT than the patient receiving amlodipine treatment. Approximately 60% of respondents believed that methadone is a type of addictive drug and that participating in MMT is a way to get high. Over 60% of respondents labeled the heroin-dependent patient who is receiving MMT as an addict even though the patient has not used heroin for several years; about 80% of them believed that the patient has undesirable characteristics and expressed a strong desire for social distance.

**Conclusions:** People's misunderstanding of MMT, and stigmatization of MMT patients were very common among populations in the communities in China. To maximize MMT patients benefiting from MMT programs, more efforts are needed to minimize the impact of MMT-related stigma.

## Introduction

### Methadone Maintenance Treatment in China

In China, opioids remain one of the most widely used illegal drugs (1), and illicit opioid use continues to drive the human immunodeficiency virus (HIV) epidemics and increase crime (2, 3). To address the severe social and medical problems associated with illicit opioid use, China initiated the methadone maintenance treatment (MMT) program with just eight sites in 2004. By the end of 2016, the program has been extended to 789 sites, with 162,000 heroin users under treatment, as stated by the annual report on drug control in China in 2017 (from the Office of China National Narcotics Control Commission, http://jhak.com/grwz/20170125.pdf). MMT has a long-established efficacy in preventing illicit opioid use, reducing crime, and decreasing the risk of HIV and hepatitis C virus (HCV) transmission (4–6).

Even though MMT is effective and widely recommended, it is still largely misunderstood and stigmatized (7–10). Negative attitudes and beliefs toward MMT are widespread, such as taking methadone is a way to get high, and patients receiving MMT are characterized as incompetent, untrustworthy, irresponsible, and has a weak willpower (11). Due to these prejudice, stereotypes, and discrimination, MMT patients have been stigmatized widely in the community (12). Research indicates that stigma against MMT gives rise to many negative consequences, including increased involvement in risky behavior, non-participation, treatment withdrawal, delayed recovery, and reintegration process (13–16).

### Stigmatization Toward MMT Patients

A previous study reported that MMT service providers had a high-level stigma toward MMT patients (17). Community-based social support may play a more crucial role in improving and continuing the treatment of individuals under MMT (18). Exploring the community population's attitudes and beliefs toward MMT and stigmatization of MMT patients in China will help us develop better MMT. Thus, the present study aimed to assess public stigmatizing attitudes toward the patients receiving MMT in China, based on Link and Phelan's conception of the stigma process. According to Link and Phelan's (19) view of stigma [stigma occurs when the following interrelated components converge: “(1) labeling: people distinguish and label human differences; (2) stereotyping: the labeled is linked to undesirable characteristics; (3) separating: the labeled is separated as an out-group; (4) the labeled experience status loss and discrimination; and (5) a power situation is necessary to allow these process to unfold]”, we hypothesize that misunderstanding of MMT and stigmatization of MMT patients would also be very common among populations in the communities in China.

## Methods

### Research Procedure and Respondents

The data was collected between December 2018 and March 2019. Participants were enrolled from an urban community in the Tianxin district of Changsha city and a rural community in the Junshan district of Yueyang city, Hunan province. Participants were randomly selected from the household-based sample in these communities by Excel worksheet. In total, 800 households were selected from the urban community with 3,363 households, and 400 households were selected from the rural community with 1,046 households. One person at least 16 years old was randomly chosen in each family. A total of 1,200 individuals were included in this study. A local organizer in each community brought interviewers into each house and gave a brief account of the survey. Respondents were provided with 10 CNY to compensate for their participation. Ethical approval was obtained from the Ethics Committee of Jiangxi Mental Hospital (No. 20180204).

### Research Instruments

In this community-based, randomized case vignette study, we developed a survey booklet, including the respondents' demography and knowledge about MMT, as well as two vignettes to assess stigmatization of MMT. Respondents in the study group received vignette 1, describing a patient receiving MMT, while respondents in the control group received vignette 2, illustrating a patient receiving amlodipine treatment. Each participant was randomly given one of the vignettes.

#### Public Knowledge About MMT

A set of questions were asked to examine the participants' knowledge about MMT, such as “In your opinion, methadone is a—(1) medicine, (2) addictive drug, (3) do not know? In your opinion, participating in MMT is a—(1) therapeutic method, (2) means of drug addiction, (3) do not know?”

#### Labeling

Participants were asked to define the person depicted in the vignette (1) normal person, (2) patient, and (3) addict with the question “In your opinion, Zhangsan is a ______.”

#### Stereotyping

The semantic differential scale was used to assess the measure of stereotyping (20). The scale comprises seven pairs of polar adjectives on seven 7-point rating scales. Scores ranged from 1 to 7, lower values indicating a more negative stereotype. Participants were categorized as having a negative stereotype about the person depicted in the vignette if they scored below 4 points.

#### Social Distance

The social distance scale (21–23) was used to assess participants' desire for social distance. Participants were asked about their willingness to engage in five forms of social contact with the person depicted in the vignette including (1) “become a neighbor,” (2) “spend an evening socializing,” (3) “make friends,” (4) “start working closely,” and (5) “become related as a family.” These items were rated on a 4-point scale from 1 (definitely) to 4 (definitely not). The mean score ranged from 1 to 4 points, with a higher score reflecting a stronger desire for social distance. Participants were viewed as unwilling to engage in these forms of interaction if they scored above the point of 2.5.

### Vignettes

We constructed one vignette depicting a patient receiving MMT and the other describing a patient receiving amlodipine treatment. Patients in the two vignettes met ICD-10 criteria for opioids dependent, currently abstinent, but receiving treatment with methadone, and hypertension, respectively.

#### Vignette 1: Patient Receiving MMT

Zhang San is a 38-year-old male. His first ingestion of heroin was at his friend's house approximately 10 years ago. In the subsequent months, the intake dosage gradually increased. He gradually lost interest in other activities in life. Because of heroin use, he lost his job. Three years ago, he initiated an attempt to stop heroin use and went to an MMT clinic to receive MMT. In the past 3 years, he has taken his medication on time and has not used heroin. He has found a job and has been working continuously since then.

#### Vignette 2: Patient Receiving Amlodipine Treatment

Zhang San is a 38-year-old male. Three years ago, he received an examination at the hospital due to occasional dizziness and lack of strength. His blood pressure was repeatedly measured over 170/100 mmHg. Zhang San was prescribed with amlodipine. In the past 3 years, he has taken his medication on time and keep his blood pressure in the normal range. He has been working continuously.

### Statistical Analysis

All statistical analyses were conducted in SPSS version 20.0 software package. The *t-*tests (for continuous variables) and χ^2^ analysis (for categorical variables) were performed to compare the differences in public stigma toward the MMT patient and the hypertension patient. Moreover, correlation analysis was performed to examine the correlation between stigmatization and the age, sex, marital status, education, resident, and monthly income of the community residents.

## Results

Of the 1,200 individuals identified in the survey, 134 (11.17%) lost contact. Thus, 1,066 (88.83%) participants were (86.00% in vignette 1 and 91.66% in vignette 2) included in this study. The demographic information is shown in Table 1. There are no differences in the sociodemographic characteristics between participants who responded to the vignette of the patient receiving amlodipine treatment and those who responded to the vignette of the patient receiving MMT.

**Table 1:** Demographic characteristics.

**Variable**	**Total (*n* = 1,066)**	**Participants respond to the vignette of the patient receiving AT (*n* = 516)**	**Participants respond to the vignette of the patient receiving MMT (*n* = 550)**	**χ^**2**^/*t***	***P***
	**M (SD)**	**M (SD)**	**M (SD)**		
Age (years)	37.38 (15.88)	37.52 (16.71)	37.25 (15.06)	*0.28*	*0.78*
	***n*** **(%)**	***n*** **(%)**	***n*** **(%)**		
Gender				1.76	0.19
Male	552 (51.8)	278 (53.9)	274 (49.8)		
Female	514 (48.2)	238 (46.1)	276 (50.2)		
Marital status				3.87	0.05
Currently married	648 (60.8)	218 (42.2)	200 (36.4)		
Not married	418 (39.2)	298 (57.8)	350 (63.4)		
Education (years)				4.91	0.19
≤ 6	44 (4.1)	14 (2.7)	21 (3.8)		
7–12	682 (63.9)	224 (43.4)	258 (46.9)		
13–17	322 (30.2)	268 (51.9)	254 (46.2)		
>18	18 (1.7)	10 (1.9)	17 (3.1)		
Income per month				4.8	0.10
≤ ¥3,000	652 (61.2)	316 (61.24)	336 (61.09)		
>¥3,000	414 (38.8)	200 (38.76)	214 (38.91)		
Residence				0.07	0.79
Urban	690 (64.7)	180 (34.9)	196 (35.6)		
Rural	376 (35.3)	336 (65.1)	354 (64.4)		

*AT, amlodipine treatment; MMT, methadone maintenance treatment. χ^2^ represents chi square value of chi-squared test for categorical variables; t represents t-value of t-tests (for continuous variables)*.

### Community Residents' Understanding of MMT

Approximately 60% of respondents believed that methadone is a type of addictive drug and that participating in MMT is a way to get high. Over 30% of the respondents expressed that they did not know what methadone is or what MMT entails. Only 10.13% of the respondents acknowledged methadone as medicine and perceived participating in MMT as a treatment method (Table 2).

**Table 2:** Community residents' understanding of methadone and MMT.

	***N***	**%**
**In your opinion, methadone is a**		
Medicine	108	10.13
Addictive drug	608	57.04
Do not know	350	32.83
**In your opinion, participating in methadone maintenance treatment is a**		
Therapeutic method	108	10.13
Way to get high	600	56.28
Do not know	358	33.58

*MMT, methadone maintenance treatment*.

### Community Residents' Stigmatization of MMT Patients

#### Labeling

More than half (64.00%) of the respondents labeled the patient receiving MMT as addicts, 18.18% identified a patient's label. Only 17.82% identified a normal label for the MMT patient, whereas 67.83% labeled the hypertension patient receiving amlodipine treatment as normal (Table 3).

**Table 3:** Community residents' stigmatization toward the patient receiving AT and the patient receiving MMT.

**Stigmatization**	**Vignettes**				***t***	**χ^**2**^**
	**The patient receiving AT (*****n*** **=** **516)**		**The patient receiving MMT (*****n*** **=** **550)**			
	**Mean (SD)**	***n* (%)^#^**	**Mean (SD)**	***n* (%)^#^**		
**Labeling**						
Normal person		350 (67.8)		98 (17.8)		256.36^*^
Patient		166 (30.6)		100 (18.2)		
Addict		0 (0.0)		352 (64.0)		
**Stereotyping**						
Good–bad	5.57 (1.21)	56 (10.9)	3.53 (1.79)	410 (74.6)		
Sincere–insincere	5.39 (1.20)	78 (15.1)	3.42 (1.74)	426 (77.5)		
Safe–dangerous	5.42 (1.22)	84 (16.3)	3.46 (1.71)	422 (76.7)		
Predictable–unpredictable	5.40 (1.20)	80 (15.5)	3.43 (1.78)	424 (77.1)		
Reliable–unreliable	5.35 (1.26)	94 (18.2)	3.56 (1.58)	426 (77.5)		
Strong–weak	5.12 (1.28)	158 (30.6)	3.57 (1.86)	398 (72.4)		
Unselfish–selfish	5.22 (1.24)	160 (20.9)	3.34 (1.78)	426 (77.5)		
Mean score	5.35 (1.12)	54 (10.5)	3.47 (1.75)	420 (76.5)	86.92^*^	210.14^*^
**Social distance**						
Become neighbor	1.71 (0.65)	56 (10.9)	2.99 (0.72)	352 (64.0)		
Become acquaintance	1.73 (0.68)	58 (11.2)	3.01 (0.73)	373 (67.7)		
Make friends	1.74 (0.65)	64 (12.4)	3.57 (0.74)	481 (87.5)		
Become co-worker	1.73 (0.65)	64 (12.4)	3.12 (0.74)	432 (78.5)		
Become family	1.74 (0.67)	64 (12.4)	3.53 (0.76)	485 (88.2)		
Mean score	1.72 (0.31)	64 (12.4)	3.33 (0.79)	450 (81.8)	81.71^*^	232.06^*^

*AT, amlodipine treatment; MMT, methadone maintenance treatment*.

# *Percentages in the part of stereotyping represent proportion of respondents who scored below than the 4 midpoint of the Semantic Differential Scale. Percentages in the part of social distance represent proportion of respondents who scored above than the 2.5 midpoint*.

* *P < 0.001*.

#### Stereotyping

The stereotypes about the patient receiving MMT were generally negative. Compared with the hypertension patient (5.35 ± 1.12), the heroin-dependent patient receiving MMT (3.47 ± 1.75) was rated far more negatively. The majority (76.46%) of respondents had a negative stereotype about the MMT patient, whereas only 10.47% had a negative stereotype about the patient receiving amlodipine treatment (Table 3).

#### Social Distance

Most of the respondents expressed a high-level desire for social distance from the patient receiving MMT. Approximately 90% of respondents were unwilling to make friends with the heroin-dependent patient receiving MMT and have the person marry into the family. Most (78.54%) were unwilling to start working closely with the person; more than half of them were unwilling to move next door to and spend an evening socializing with the person. Respondents showed a higher level of desire for social distance from the patient receiving MMT (3.33 ± 0.79) than the patient receiving amlodipine treatment (1.72 ± 0.31). More details are shown in Table 3.

Correlation analysis did not find any associations between sociodemographic with the views about MMT, negative stereotype, or social intolerance toward the patient receiving MMT (as shown in the Supplementary Material).

## Discussion

As far as we know, this is the first study to evaluate the misunderstanding of MMT and stigmatization toward MMT patients in China. Findings of this study reveal that misunderstanding of MMT is widely spread in the community in Hunan, China. Although MMT effectively reduces opioids use, criminality, and adverse health effects, only 10.13% of the respondents acknowledged methadone as medicine and perceived MMT as a treatment method. Consistent with reports that MMT continues to be largely misunderstood (7, 11, 12), many respondents perceived methadone as a type of addictive drug and that participating in MMT is a way to get high.

### High Level of Stigma and Discrimination Toward MMT Patients

In these two vignettes, respondents showed a significantly higher level of stigma and discrimination toward patients receiving MMT than patients receiving amlodipine treatment. Approximately 60% of respondents believed that methadone is a type of addictive drug and that individuals participate in MMT to get high. Over 60% of respondents labeled the heroin-dependent patient who is receiving MMT as an addict. About 80% believed the patient has undesirable characteristics and expressed a strong desire for social distance.

These findings also show stigmatization of MMT patients compared with patients with other chronic conditions that require treatment such as hypertension. The majority of the community respondents in the community labeled the methadone patient as an addict, believing the patient receiving MMT is bad, insincere, dangerous, unpredictable, unreliable, weak, and selfish. Since negative stereotype could evoke community rejection (24), it is no surprise that the respondents expressed a strong desire for social distance across different domains of social interaction with patients receiving MMT. Participants expressed that the more intimate the interaction, the stronger the desire to keep a distance. Thus, the vast majority were unwilling to make friends with the patient receiving MMT or have the person marry into the family.

These findings in our study suggest that widespread misunderstanding of MMT and stigmatization of the patients in the community should be taken into account in the community-based MMT program. China has established the largest MMT network in the world to address the serious social and medical problems associated with illicit opioid use. However, the present study indicates that community residents are far from being well-prepared to accept methadone patients. The continuing stigmatization of methadone patients may undermine the government's efforts to help them tackle their condition. Interventions to resolve misunderstandings and reduce stigmatization are necessary for all sections of the community.

Many studies have demonstrated that the MMT stigma is an extension of substance use stigma (8, 11, 25–28). Therefore, interventions that reduce MMT stigma may need to co-address substance use stigma (27). Keeping a reasonable balance between raising public awareness of drugs while avoiding discrimination and rejection against drug users is an important issue in public media communication. Previous evidence demonstrated that communicating with the public using sympathetic stories or narratives that humanize the experiences of individuals with substance use disorder is a promising technique for reducing stigma (29–31). In contrast, messages blaming individuals with substance use disorder will increase public negative emotions and desire for social distance toward them (32, 33). Furthermore, information about the therapeutic quality of MMT is especially needed to address stigma. Stories or narratives portraying individuals with successful treatment recovery would reduce MMT stigma (34–36).

### Limitations

There are several limitations to the study. First, this study is based on self-report data which may induce self-report bias. Second, information was obtained through closed-ended questions, which may guide participants to provide socially desirable responses. Third, other factors other than MMT depicted in the vignette, such as heroin dependence, may impact responses, thus leading to overestimated stigma levels. Fourth, participants were recruited from two communities in Hunan, which may not fully represent all community members in this province or in China. Despite this, participants were randomly selected from these two communities, which reduced bias and increase the representation of findings. Fifth, as 11.17% of the identified individuals could not be contacted, it is unknown if their views and attitudes toward MMT patients are different from the respondents. Sixth, the vignette response rate described that patient receiving MMT (86.00%) was much lower than the patient receiving amlodipine treatment (91.66%). These non-responders who received MMT vignette may have more negative attitudes toward patients receiving MMT, which may lead to underestimation of the stigmatization toward MMT. Yet, the present result still reflects a high level of stigmatization attitudes toward MMT and patients receiving MMT in community residents. Last but not least, we did not evaluate participants' personal experience, such as having encountered such patients in the past, which may influence results and could provide some information about prevention and intervention.

## Conclusions

Our findings demonstrate that misunderstanding of MMT and stigmatization of MMT patients are very common among populations in the communities in China. Educating community residents regarding MMT's therapeutic quality and the patient identity of the person receiving MMT is recommended.

## Data Availability Statement

The raw data supporting the conclusions of this article will be made available by the authors, without undue reservation.

## Ethics Statement

The studies involving human participants were reviewed and approved by Ethic Committee of Jiangxi Mental Hospital. Written informed consent to participate in this study was provided by the participants' legal guardian/next of kin.

## Author Contributions

TL and QW performed the statistical analysis. QD, QW, and TL prepared the first draft of the manuscript. QD, MH, FY, and QL assisted in the collection of data and implementation. QD, TL, and QW have contributed to the interpretation of data and writing. WH commented on the manuscript. All authors participated in the research, study design, manuscript preparation, and have approved the final manuscript.

## Conflict of Interest

The authors declare that the research was conducted in the absence of any commercial or financial relationships that could be construed as a potential conflict of interest.
