# Supplementary material for: A Community-Based Investigation of Stigma Toward Individuals Receiving Methadone Maintenance Treatment in China: A Randomized Case Vignette Study
Source: Front Psychiatry. 2020 Nov 25;11:601266. doi: 10.3389/fpsyt.2020.601266 (PMC7723969; doi:10.3389/fpsyt.2020.601266)
Supplement: Supplementary file 1 [file Table_1.DOC]

**Supplementary Table 1. Correlations of demographic characteristics and community residents’ understanding of methadone and MMT (n = 1066)**

|  | Age | Gender | Marital status | Education | Income per month | Residence |
| --- | --- | --- | --- | --- | --- | --- |
| Understanding of methadone | -0.035 | -0.013 | -0.063 | 0.012 | -0.012 | 0.006 |
| Understanding of MMT | -0.036 | -0.003 | -0.064 | 0.024 | -0.001 | 0.002 |

*Note:* MMT: methadone maintenance treatment. All P values were more than 0.05.

**Supplementary Table 2. Correlations of demographic characteristics and community residents’ stigmatization towards the patient receiving MMT (n = 550)**

|  | Age | Gender | Marital status | Education | Income per month | Residence |
| --- | --- | --- | --- | --- | --- | --- |
| Labeling | -0.004 | 0.026 | 0.066 | -0.049 | -0.093 | 0.033 |
| Stereotyping | -0.009 | 0.008 | -0.008 | -0.006 | 0.06 | 0.08 |
| Social distance | 0.026 | -0.06 | -0.03 | 0.018 | 0.003 | 0.009 |

*Note:* MMT: methadone maintenance treatment. All P values were more than 0.05.
